# Gradual Thickening of the Peritubular Lamina Propria in Healthy Boar Seminiferous Tubules Due to Cryptorchidism: Increased Immunoexpression of Diverse Proteins in Sertoli and Myoid Cells

**DOI:** 10.3390/ani15121696

**Published:** 2025-06-08

**Authors:** Vicente Seco-Rovira, Jesús Martínez-Hernández, Laís Freire-Brito, Ester Beltrán-Frutos, Juan Francisco Madrid, Luis Miguel Pastor

**Affiliations:** 1 Department of Cell Biology and Histology, Medical School, Biomedical Research Institute of Murcia Pascual Parrilla–IMIB, Regional Campus of International Excellence “Campus Mare Nostrum”, University of Murcia, 30120 Murcia, Spain; vicente.seco@um.es (V.S.-R.); jesus.martinez7@um.es (J.M.-H.); ebf96527@um.es (E.B.-F.); jfmadrid@um.es (J.F.M.); 2 Clinical and Experimental Endocrinology, UMIB-Unit for Multidisciplinary Research in Biomedicine, ICBAS-School of Medicine and Biomedical Science, University of Porto, 4050-313 Porto, Portugal; laisbrito0330@gmail.com; 3 Laboratory for Integrative and Translational Research in Population Health (ITR), University of Porto, 4200-465 Porto, Portugal

**Keywords:** testes, cryptorchidism, Sertoli, myoid cell, extracellular matrix, peritubular lamina propia

## Abstract

In testicular pathology, the accumulation of extracellular matrix and thickening of the peritubular lamina propria are associated with altered spermatogenesis. This study evaluated the immunoexpression of certain proteins in Sertoli and myoid cells of cryptorchid boar seminiferous tubule sections to determine the role of these cells in peritubular lamina propria thickening. Sections of normal seminiferous tubules and those with varying degrees of atrophy (I–III) were studied using histochemical and immunohistochemical techniques. A semiquantitative analysis of histochemical staining or immunostaining intensity in these sections was performed. Gradual thickening of the peritubular lamina propria was observed between stage II and III sections. HSP47 was present in myoid and Sertoli cells in normal sections and was significantly increased in stage II and III sections. The increase in stage II was dependent on Sertoli cells, while in stage III, it depended on myoid cells. The vimentin increases in stage I sections were due to Sertoli cells and later to myoid cells in stages II and III. α-actin and collagen IV immunoreactivity were observed from the early stage of atrophy, with a significant increase in stage III. In conclusion, in porcine spontaneous cryptorchidism, the seminiferous tubule exhibits gradual fibrotic alterations in the peritubular lamina propria, initially related to changes in the Sertoli cell phenotype and later, in the final stages, to myoid cells. Collagen I deposition appears to be caused by myoid cells, initiating the sclerosis of the seminiferous tubules.

## Simple Summary

Thickening of the peritubular lamina propria surrounding the seminiferous tubules is linked to altered spermatogenesis. Herein, we analyzed protein expression in Sertoli and myoid cells from the seminiferous tubules of healthy, postpubertal, cryptorchid boars to better understand their role in this thickening. Several degrees of atrophy were observed in the tubules, and a thickening of the peritubular layer was found as atrophy progressed. HSP47 protein expression increased in Sertoli and myoid cells as atrophy progressed. Increases in other proteins, such as vimentin, α-actin, and collagen IV, were also noted, especially in the more advanced stages of atrophy. In summary, in boar cryptorchidism, changes in Sertoli and myoid cells contribute to the thickening and fibrosis of the seminiferous tubules.

## 1. Introduction

The accumulation of extracellular matrix (ECM) and thickening of the peritubular lamina propria (PLP) are associated with impaired spermatogenesis [1,2,3], as occurs in aged seminiferous tubules and patients with various testicular pathologies. This phenomenon is observed in varicocele [4], Klinefelter’s syndrome [5,6], after long-term estrogen treatment [7], Sertoli cell-only syndrome [8], and in azoospermic and oligozoospermic patients [9,10]. The thickening of the PLP appears to be due to increased deposition of collagen fibrils in the extracellular space, resembling the fibrosis observed in other organs, which involves increased numbers of fibroblasts/myofibroblasts and excessive deposition of ECM proteins [11]. The explanation for this fibrosis is unclear, although some authors suggest that the dedifferentiation of myofibroblasts with loss of their contractile markers is related to PLP thickening [12]. Other authors argue that myofibroblast dedifferentiation is not responsible for the disturbed spermatogenesis associated with PLP alterations. Myofibroblasts, presumably generated de novo in part, might contribute to disturbed spermatogenesis as key players during the development of fibrotic alterations in PLP, but not through contractile dysfunction in these cells [3]. There is considerable debate in testicular histopathology about whether this phenomenon directly causes the deterioration of spermatogenesis or if it is instead a final consequence of previous pathological alterations [13]. Currently, it can be stated that there is a negative correlation between PLP thickness and the degree of spermatogenesis, indicating that a thin PLP may be a sign of normal spermatogenesis [13]. Moreover, it can be concluded that fibrotic PLP changes are a common pathological reaction pattern of the testis rather than a disease-specific one [3].

The above-mentioned phenomenon has also been observed in human cryptorchidism. The PLP of seminiferous tubules is affected at an early age [14], and its pathological changes over time involve progressive thickening of the inner and outer acellular layers due to an accumulation of collagen fibers and microfibrillar material [2,15]. The basement membrane (BM) also thickens and appears homogeneous or multilaminar [16]. Additionally, modifications of Sertoli and peritubular myoid cells in cryptorchidism are important; changes in intermediate filaments could induce functional changes in Sertoli cells, partially contributing to germ cell apoptosis leading to azoospermia or oligozoospermia [17]. Enhanced Sertoli cell degeneration in cryptorchid testes must also contribute to the reduction in germ cell number [18], and diverse states of Sertoli cell maturation are related to varying degrees of seminiferous epithelium deterioration [15]. In boar testes, the PLP, unlike laboratory animals, has two layers of myofibroblasts separated by ECM [19]. The study of spontaneous postpubertal cryptorchidism in boars—unilateral and bilateral—seems useful because the results can be extrapolated to humans [19,20,21] to gain a better understanding of the pathogenesis of adult cryptorchid males [22,23,24]. The morphological characteristics of boar PLP in cryptorchidism show gradual histological changes from seminiferous tubules with spermatogenesis arrest to partial and complete tubular hyalinization. This may be related to a lack of tubular contractility due to a fibroblastic phenotype of peritubular cells. Moreover, the severity of these abnormalities seems to correlate with the degree of damage to Sertoli cells [19].

Heat shock protein 47 (HSP47) is an essential protein involved in the correct folding of collagen; it is a glycoprotein belonging to the serpin superfamily, also known as serpinH1. HSP47 is a collagen-specific chaperone located in the endoplasmic reticulum (ER) and its main function is collagen maturation, interacting specifically with procollagen molecules [25]. In aging and diverse testicular pathologies, the phenomenon of deposition of extracellular material in the PLP is driven by both myoid and Sertoli cells [3,13], with increases in laminin, fibronectin, and collagen I or IV in this structure being associated with impaired spermatogenesis [2,3,26,27]. Recent studies have shown a link between increased HSP47 expression and excessive collagen accumulation in scar tissues in various human fibrotic diseases [28,29] and is thus considered a potential target in fibrotic diseases [30]. In mouse testis, HSP47 was first identified in association with collagen XXVI synthesis during the neonatal period [31], suggesting the presence of this chaperone in myoid cells. In addition, a gene expression study in rat Leydig cells during aging found overexpression of HSP47 protein transcripts in aged rats with regressed testes compared with non-regressed [32]. HSP47 was also detected in fibroblast cells of vasectomized rats with fibrosis of the testicular interstitium [33]. More recently, a higher percentage of HSP47-positive Leydig cells was detected immunohistochemically in Syrian hamster testes exposed to a short photoperiod compared with a long one [27].

For the first time in mammals, our research group recently identified the presence of HSP47 in the Sertoli cells of the Syrian hamster [34]. Semiquantitatively, we observed HSP47 overexpression in Sertoli cells of aged hamster seminiferous tubules with spermatogenesis arrest compared with those tubules in hypospermatogenesis or with normal spermatogenesis. This overexpression has been related to a thickened PLP in ageing, hypothesizing that, in various testicular pathologies, seminiferous tubule sclerosis and hyalinization must be accompanied by new collagen synthesis by both myoid and Sertoli cells during the process of PLP thickening following impaired spermatogenesis [34].

In the present work, based on these recent findings, we aimed to evaluate the common behavior of Sertoli and myoid cells in healthy testes and postpubertal spontaneous boar cryptorchidism. We analyzed the expression of HSP47 and other proteins of the cellular differentiation state of these cells to determine their involvement in the ECM deposition responsible for the progressive thickening of the PLP.

## 2. Materials and Methods

### 2.1. Animals and Tissue Preparation

The study was conducted using four postpubertal male *Sus domesticus* (used in previous studies by our research group [19,24]). The testes were categorized into three experimental groups: unaffected testis of a healthy boar, unilateral cryptorchid testis, and bilateral cryptorchid testis. The animals were maintained in a controlled environment at an average temperature of 18 ± 1 °C and fed a nutritious diet. The boars were slaughtered at nine months of age, and the testes were immediately removed and processed for light microscopy examination. Briefly, samples were fixed in methacarn (methanol: chloroform: acetic acid 6:3:1), dehydrated through an increasing ethanol series, and embedded in paraffin wax at 56–58 °C. Subsequently, 5 µm-thick sections were cut on a rotary microtome (Leica Biosystems)Nussloch, Germany) [24]. This study was conducted in accordance with Spanish ethical and legal standards regarding animal protection.

### 2.2. Histochemical Study and Immunohistochemical Identification of PLP-Related Proteins

For the histochemical study of the tubular wall, picrosirius red (PSR) and Alcian blue (pH = 2.5) (AB) staining techniques were performed [35,36]. For the immunohistochemical study, the proteins analyzed were HSP47, vimentin, α-actin, and collagen IV (Col IV). Briefly, samples were dewaxed in xylol and rehydrated in an ascending ethanol gradient. For HSP47, antigen retrieval was performed using an antigen unmasking buffer (Agilent Dako; Santa Clara, CA, USA, IS611, S2367) in a 95 °C water bath for 40 min. Antigen retrieval was not necessary for the other proteins. After antigen retrieval, samples were rinsed with phosphate-buffered saline (PBS), the peroxidase activity was blocked, and samples were incubated overnight at 4 °C with each primary antibody at the appropriate dilution [polyclonal rabbit anti-HSP47 antibody at 1:100 dilution (Santa Cruz Biotechnology; Dallas, TX, USA, sc-8352), monoclonal mouse anti-α-actin (Clone 1A4) (Agilent Dako; Santa Clara, CA, USA, IS611), monoclonal mouse anti-vimentin (Clone V9) antibody at 1:50 dilution (Agilent Dako; GA630), and monoclonal mouse anti-collagen IV antibody at 1:50 dilution (Agilent, Dako, M0785)] in 1% PBS/bovine serum albumin (BSA). Samples were then rinsed with PBS and incubated with the appropriate biotinylated secondary antibody for 45 min at room temperature in a 1:300 solution in 1% PBS/BSA. After incubation with peroxidase-conjugated streptavidin (DAKO; P0397) in a 1:300 solution in 1% PBS/BSA for 30 min at room temperature, samples were rinsed with PBS. The antibody–peroxidase complex was developed using PBS containing 0.05% diaminobenzidine (DAB) (Sigma Aldrich Subsidiary of Merck KGaA, Burlington, MA, USA) and 0.3% H_2_O_2_ for three minutes at 18–24 °C. Finally, sections were washed in fresh water, counterstained with hematoxylin for 10 s, dehydrated through ascending grades of alcohol, and mounted in dibutyl phthalate polystyrene xylene (DPX) (Merck Darmstadt, Germany). The specificity of the immunohistochemical techniques was confirmed on sections incubated with non-immune serum instead of the primary antibody [34].

### 2.3. Histochemical Semiquantitative Analysis of PLP in Seminiferous Tubule Atrophy Due to Cryptorchidism

Ten cross-sectioned seminiferous tubule sections of non-cryptorchid testes and each stage of testicular atrophy in cryptorchid testes—I, II, and III—were selected. The area of the tubular lumen was not considered in this analysis. A semiquantitative analysis assessed the relative amount of peritubular collagen, expressed as the PSR staining area (PSR-SA) relative to the seminiferous tubule area, including the PLP, (STA). Thus, the ratio between both parameters was obtained as (PSR-SA/STA) ×100 in each of the tubular sections studied [34]. Additionally, four measurements of PLP thickness were taken at opposite poles of each tubule section to assess PLP thickening. The AB histochemical technique was used to study the type of glycoconjugates in the tubular wall. The groups studied and the number of sections were the same as in the previous analysis. The area of the tubular lumen was eliminated in this analysis. A semiquantitative analysis of the nature of the tubular wall components was conducted by measuring the positive staining area for AB (AB-SA) relative to the STA. Thus, a ratio between both parameters was obtained, expressed as (AB-SA/STA) ×100 in each atrophy stage studied [34]. These areas were determined using an Olympus BX51 light microscope (Olympus, Hicksville, NY, USA) with an Olympus DP 25 camera attached (Olympus, Hicksville, NY, USA). Images were analysed with Cell D image analysis software GmbH (Olympus, Hicksville, NY, USA). Before the study, the intensity of both PSR and AB staining was determined in the four types of tubule sections as a reference for image acquisition.

### 2.4. Semiquantitative Analysis of HSP47, Vimentin, α-Actin, and Collagen IV Expression in Relation to Seminiferous Tubule Atrophy Due to Cryptorchidism

For this study, the seminiferous tubule section types were the same as in the study of the amount of ECM in the PLP. For each study group, 10 tubule sections were selected for each immunohistochemical staining. A semiquantitative study of each protein immunoreactivity was performed in all stages of tubular atrophy. The area of the tubular lumen was not considered in these analyses. To this end, the area of immunoreactivity to HSP47 (AIR-HSP47), Vimentin (AIR-Vim), α-actin (AIR-αAc), and Col IV (AIR-Col IV), and STA, including the PLP, were calculated for each section; the AIR/STA ratio was obtained as (AIR/STA) ×100 [34]. For HSP47 and vimentin, the immunoreactive area of the PLP and seminiferous epithelium was also calculated separately. The microscope, digital camera, and image analysis software were the same as in the previous section. Before the study, the intensity of HSP47, vimentin, Col IV, or α-actin positivity was determined in the four types of tubule sections as a reference for image acquisition.

### 2.5. Statistical Analysis

Equality of means was tested by one-way analysis of variance (ANOVA) followed by a post hoc test of equality between pairs of means using the least significant difference (LSD) and Bonferroni methods, considering the total number of animals and the number of components in each group. Results were considered statistically significant when the *p*-value was below 0.05. A bilateral correlation analysis was also performed for each parameter studied. The statistical software package SPSS 28 (IBM, Madrid, Spain) was used.

## 3. Results

### 3.1. Histological Characterization of the Degree of Testicular Atrophy Due to Cryptorchidism

**Figure 1:**
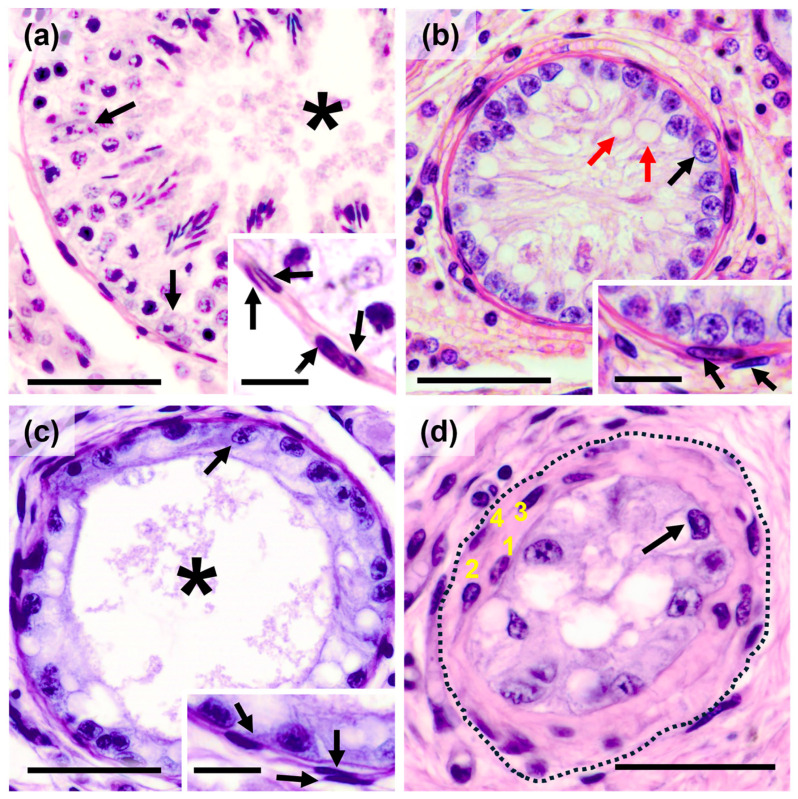
Hematoxylin–Eosin: (**a**) Section of a seminiferous tubule with complete spermatogenesis, where Sertoli cells with normal morphology (arrows) and a wide tubular lumen (asterisk) were observed. The insert detailed the composition of the tubular wall, made up of two layers of myoid cells (arrows). (**b**) Tubule section in stage I atrophy where, in addition to the considerable reduction in tubule diameter, only Sertoli cells (black arrows) with large cytoplasmic vacuolations (red arrows) and a total absence of the tubular lumen was observed. The tubular wall maintained two layers of peritubular myoid cells (arrows). (**c**) Sertoli cell retraction (arrow) was observed in stage II tubular atrophy, leaving a large tubular lumen (asterisk). At this stage, the tubular wall was formed by three layers of peritubular myoid cells. (**d**) In stage III atrophy, the seminiferous tubule had a significantly reduced diameter (dashed line) and was formed by a small number of Sertoli cells (arrow) with no appreciable tubular lumen. Additionally, the tubular wall appeared significantly thickened and formed by four layers of peritubular cells (1 to 4). Scale bar: (**a**–**d**) 50 µm. Insert scale bar: 25 µm.

The seminiferous tubule sections from cryptorchidic testes were classified into three types, ranging from least to most affected, reflecting different grades or stages of cryptorchidic atrophy [19]. These were compared with normal tubule sections from healthy animals, where spermatogenesis is complete, and the peritubular myoid cells of the tubular wall maintain a normal histological two-layer conformation (Figure 1a and Table 1).

Stage I: The seminiferous tubules are solely composed of Sertoli cells with large cytoplasmic vacuolations and some spermatogonia, and the tubular lumen is practically non-existent. Furthermore, the peritubular cells are distributed into two layers. (Figure 1b).

Stage II: The tubules are composed only of Sertoli cells that maintain the large cytoplasmic vacuolations, but their cytoplasmic extensions are retracted, leaving a large tubular lumen inside. The peritubular cells form a three-layer pattern, with the appearance of cells with larger and more heterochromatic nuclei in some cases. Additionally, a slight thickening of the PLP is observed (Figure 1c).

Stage III: The seminiferous tubules are in a very advanced state of atrophy, where only some Sertoli cells with large cytoplasmic vacuolations are observed, and the tubular lumen is practically non-existent. The PLP is very thickened, and up to four layers of peritubular cells can be observed: an outermost layer with fibroblastic cells with thin, spindle-shaped nuclei, two intermediate layers with myoid cells with slightly larger, more oval nuclei than the previous layer, and an innermost layer with few cells with more rounded nuclei (Figure 1d).

### 3.2. Semiquantitative Histochemical Evaluation of PLP Components During Seminiferous Tubule Atrophy in Cryptorchidism

**Figure 2:**
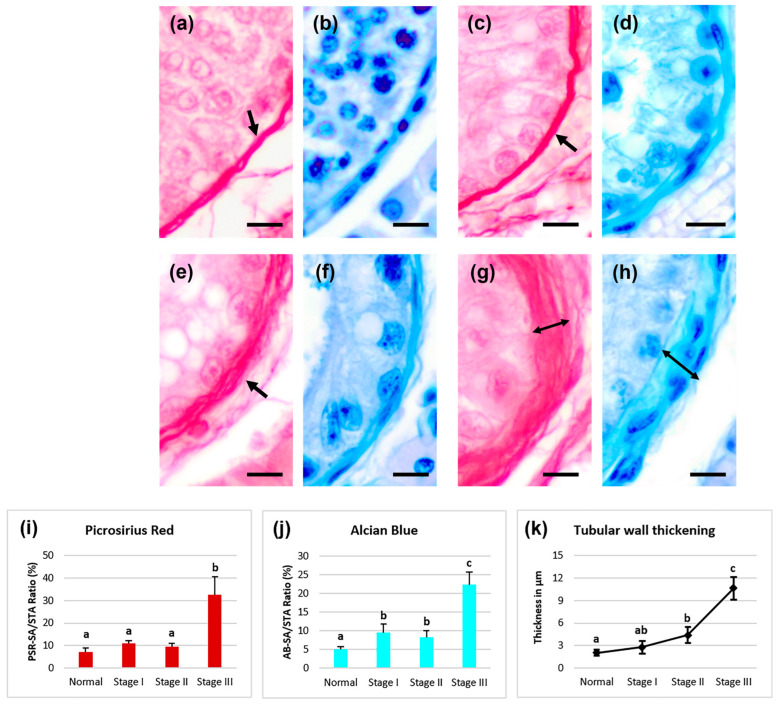
Picrosirius Red (PSR) (**a**,**c**,**e**,**g**) and Alcian Blue pH 2.5 (AB) (**b**,**d**,**f**,**h**) histochemical staining. (**a**,**b**) In normal tubules, staining of the tubular wall (arrow) was observed with PSR and AB (**b**). (**c**,**d**) Stage I. PSR staining was very similar to the normal stage (**c**), while with AB, the staining intensity was significantly greater (**d**). (**e**,**f**) In stage II atrophy, a thickening of the tubular wall (arrow) was observed compared with the previous groups (**e**,**k**). The PSR-SA/STA ratio did not increase significantly (**i**). Likewise, the AB-SA/STA ratio remained similar to the previous group (**f**,**j**). (**g**,**h**) In stage III atrophy, there was a significant increase in the PSR-SA/STA and AB-SA/STA ratios (**i,j**), as well as in tubular wall thickness (double arrow bar, **k**). a, b, and c showed significant differences (*p* < 0.05). Scale bar: 10 µm.

No significant increase in the collagen PSR-SA/STA ratio was observed between seminiferous tubule sections with normal histology (Figure 2a) and those in stage I and II atrophy (Figure 2c,e). The collagen ratio rose significantly higher (*p* < 0.05) in sections in stage III tubular atrophy (Figure 2g,i). Additionally, this technique evidenced the tubular wall thickening in the different stages of seminiferous tubule atrophy. There was statistically significant thickening (*p* < 0.05) from stage II atrophy onwards, becoming more pronounced in stage III (Figure 2k).

The AB technique revealed the nature of the acidic glycoconjugates in the tubular wall. This method highlighted the evolution of cell wall components from a histochemical perspective, starting with normal tubules (Figure 2b) and continuing through all the previously described stages of tubular atrophy (Figure 2d,f,h). The AB-SA/STA ratio presented a significant increase (*p* < 0.05) in polysaccharide compounds rich in acid groups throughout the cryptorchidism-induced testicular atrophy process; this increase was much higher (*p* < 0.05) in stage I and III tubular atrophy (Figure 2j).

### 3.3. Semiquantitative Immunohistochemical Evaluation of PLP in Seminiferous Tubules During Testicular Atrophy in Cryptorchidism

The immunohistochemical study of the different atrophy stages revealed variations in the expression of proteins characteristic of the PLP and Sertoli or myoid cells.

#### 3.3.1. Heat Shock Protein 47

**Figure 3:**
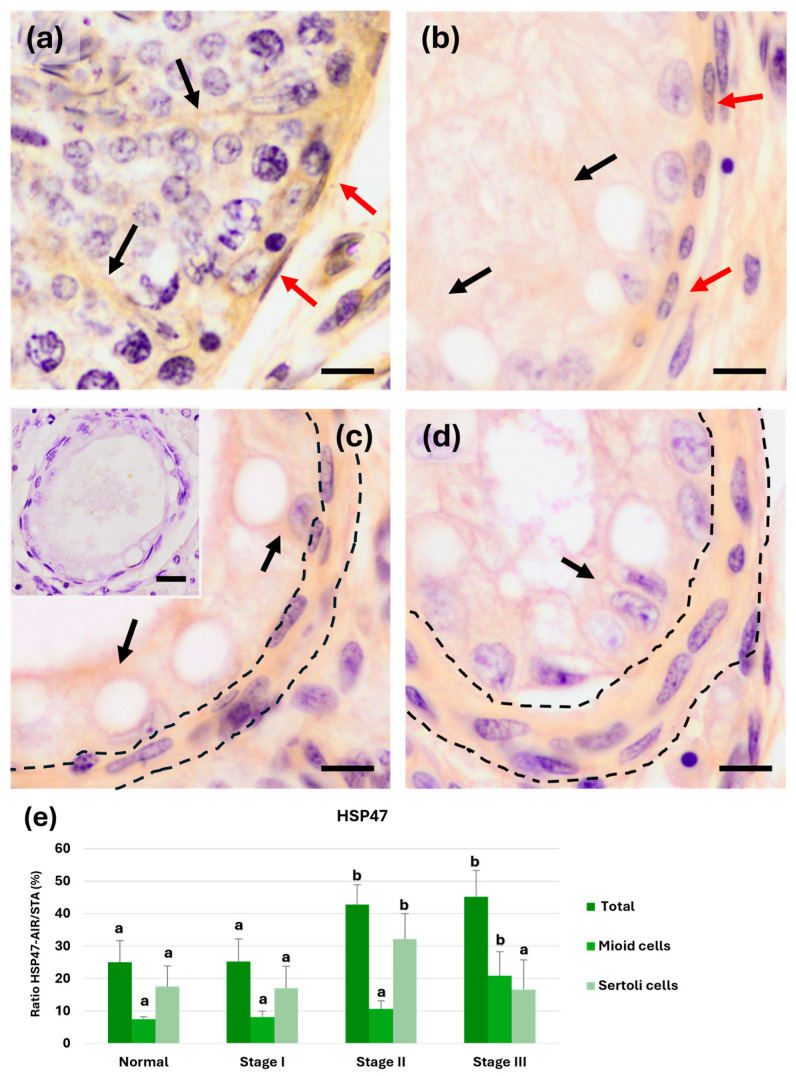
HSP47 immunohistochemistry AIR-HSP47). (**a**) Normal tubules. AIR-HSP47 was observed in the extensions of both Sertoli (black arrows) and myoid cells (red arrows). (**b**) In stage I atrophy, HSP47 immunoreactivity remained similar in both Sertoli (black arrows) and myoid cells (red arrows). (**c**) In stage II, the HSP47 immunoreactivity area increased in Sertoli cells (arrows) despite the greater thickness of the tubular wall (dotted lines). (**d**) In stage III, the HSP47 immunoreactivity area decreased in Sertoli cells (arrow) and increased in the myoid cells of the wall (dotted lines). (**e**) Graph showing the evolution of HSP47 expression generally and in Sertoli and myoid cells. a and b show significant differences (*p* < 0.05). Scale bar: 10 µm. Insert: negative control with anti-rabbit secondary antibody, scale bar: 20 µm.

HSP47 immunoreactivity was observed in Sertoli and myoid cells of the PLP in normal tubule sections (Figure 3a) and throughout the gradual process of atrophy (Figure 3b–d). The ratio between the area of HSP47 immunoreactivity (AIR-HSP47) and STA increased in stages II and III compared with stage I and normal tubules (*p* < 0.05). AIR-HSP47 was also specifically calculated in the PLP and the epithelium observed in Sertoli cells. Across the different atrophy stages in seminiferous tubule sections, HSP47 immunoreactivity in myoid cells remained similar to the control in stages I and II atrophy. However, a significant increase (*p* < 0.05) was observed in stage III atrophy compared with the other stages and normal tubules (Figure 3). In contrast, in Sertoli cells, the significant increase only occurred in stage II (*p* < 0.05). In summary, an increase in HSP47 expression appears when reaching stage II atrophy, primarily caused by Sertoli cells. However, in stage III, the increased HSP47 expression is driven by peritubular myoid cells (*p* < 0.05) (Figure 3e).

#### 3.3.2. Vimentin

**Figure 4:**
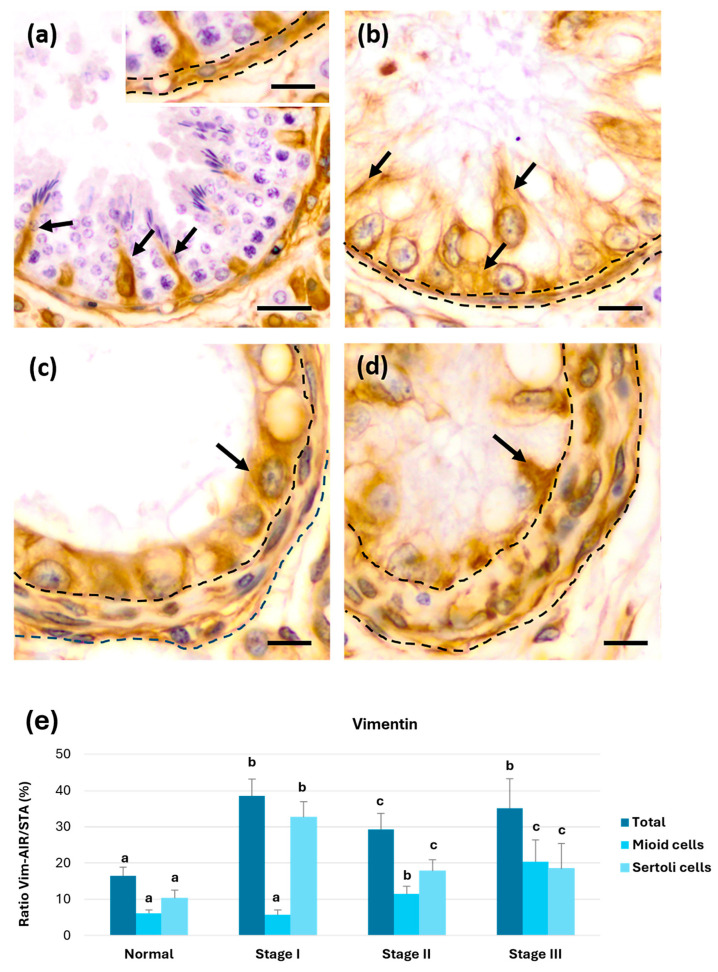
Immunohistochemical staining for vimentin (Vim). (**a**) Normal tubules showing Sertoli (arrows) and myoid cells of the tubular wall (insert, dotted lines) with well-defined expression. (**b**) In stage I atrophy, strong immunostaining was observed in Sertoli cells (arrows), while in the tubular wall, it remains similar to the control (dotted line). (**c**) In stage II, positivity was observed near the nuclei of Sertoli cells (arrow), along with an increase in tubular wall thickness (dotted line). (**d**) In stage III, Vim expression in Sertoli cells remained stable (arrows), while tubular wall of myoid cells increased their immunoreactivity (dotted line). (**e**) Graph showing the evolution of Vim expression both total and in Sertoli and myoid cells. a, b, and c show significant differences (*p* < 0.05). Scale bars: (**a**) 25 µm; insert (**a**–**d**) 10 µm.

Vimentin immunostaining in normal seminiferous tubule sections was observed both in Sertoli and myoid cells of the PLP (Figure 4a). In stage I atrophy, vimentin immunoreactivity in Sertoli cells appeared to increase; nonetheless, no apparent changes were observed in myoid cells (Figure 4b). In stages II and III, a change in the immunostaining pattern was noted due to variations in Sertoli cell morphology. Additionally, the number of vimentin-positive myoid cells appeared to increase (Figure 4c,d).

The ratio between the area of vimentin immunoreactivity (Vim-AIR) and STA showed a significant increase (*p* < 0.05) in the early stages of tubular atrophy (stage I). However, it significantly decreased in stage II tubules compared with stages I and III, then increased again in stage III. Vim-AIR was also calculated specifically in the PLP (positivity in myoid cells) and the epithelium observed in Sertoli cells. In the PLP (myoid cells), there was a significant increase (*p* < 0.05) in immunoreactivity in tubule sections with stage II atrophy compared with normal sections, which continued to increase significantly (*p* < 0.05) in stage III atrophy sections. In the epithelium (Sertoli cells), a significant increase (*p* < 0.05) in vimentin immunoreactivity was observed in early atrophy (stage I) compared with normal tubules. In stage II and III atrophy, vimentin immunoreactivity significantly decreased (*p* < 0.05) compared with stage I but remained higher than in normal tubule sections.

In summary, the initial increase in vimentin was driven by its expression in Sertoli cells, which significantly decreased as the degree of tubular atrophy increased (stages II and III). However, this decrease was compensated by a strong increase in vimentin expression in peritubular myoid cells in tubules with stage II and III atrophy (Figure 4e).

#### 3.3.3. α-Actin in PLP Myoid Cells

**Figure 5:**
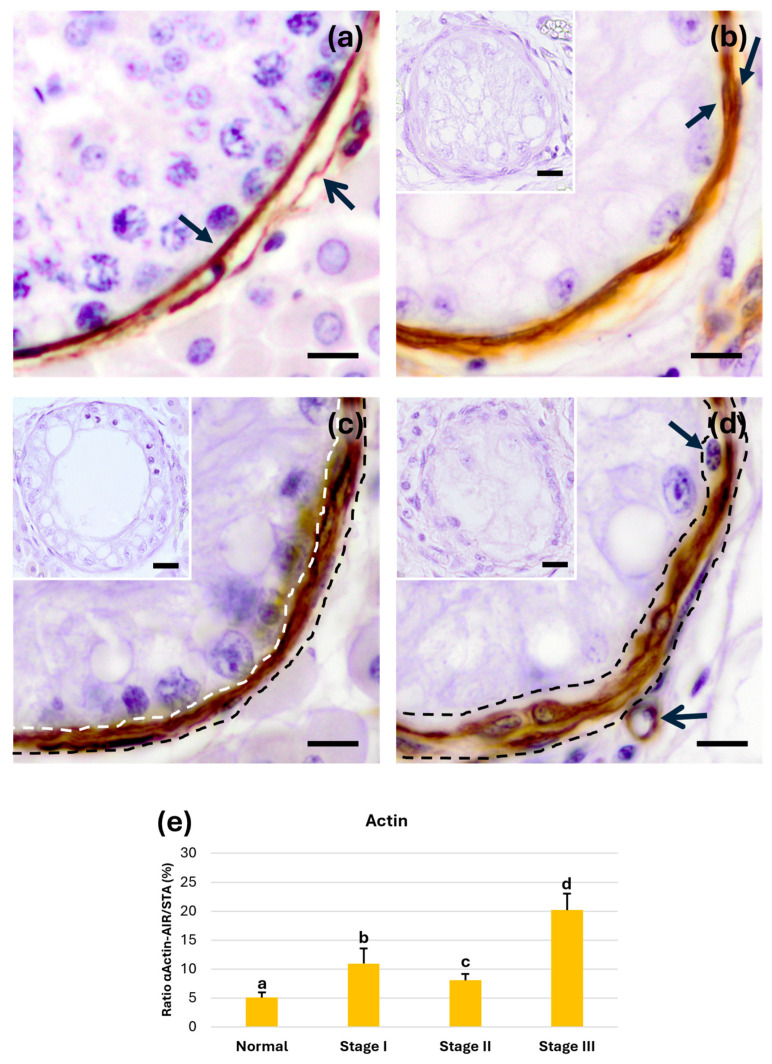
Immunohistochemistry for α-actin. (**a**) α-actin immunoreactivity pattern in the normal group showing positivity both in myoid cells of the tubular wall (closed arrow) and in the walls of surrounding vessels (open arrow). (**b**) In stage II, all cells of the tubular wall were positive with this technique (arrows). (**c**) In stage II, tubular wall thickening was observed (white and black dotted lines). (**d**) Finally, in stage III, within the cells forming the tubular wall, the innermost cells were negative for α-actin (arrow), showing positivity in other cells comprising the wall and surrounding blood vessels (open arrow). (**e**) Semiquantitative results expressed as the α-Ac-AIR/STA (%) ratio of α-actin immunoreactivity during the different stages of tubular atrophy. a, b, c, and d show significant differences (*p* < 0.05). Scale bar: 10 µm. Inserts: negative control with anti-mouse secondary antibody scale bar: 20 µm.

The staining intensity was similar across all groups; however, the α-actin immunoreactivity area in myoid cells increased from the control group (Figure 5a). In stage I tubular atrophy (Figure 5b), the expression pattern was very similar to the control group, while thickening of the PLP was noted in stage II (Figure 5c). Finally, in stage III tubular atrophy (Figure 5d), not all cells forming the tubular wall exhibited the same degree of α-actin positivity.

From a semiquantitative perspective, a significant increase (*p* < 0.05) in the α-actin immunoreactivity ratio was observed in stage I tubular atrophy compared with normal tubules. The immunoreactivity decreased significantly (*p* < 0.05) in stage II atrophy tubules compared with stages I and III and then significantly increased again (*p* < 0.05) in tubules with stage III atrophy (Figure 5e).

#### 3.3.4. Immunoexpression of Collagen IV in the Basement Membrane of Seminiferous Tubules

**Figure 6:**
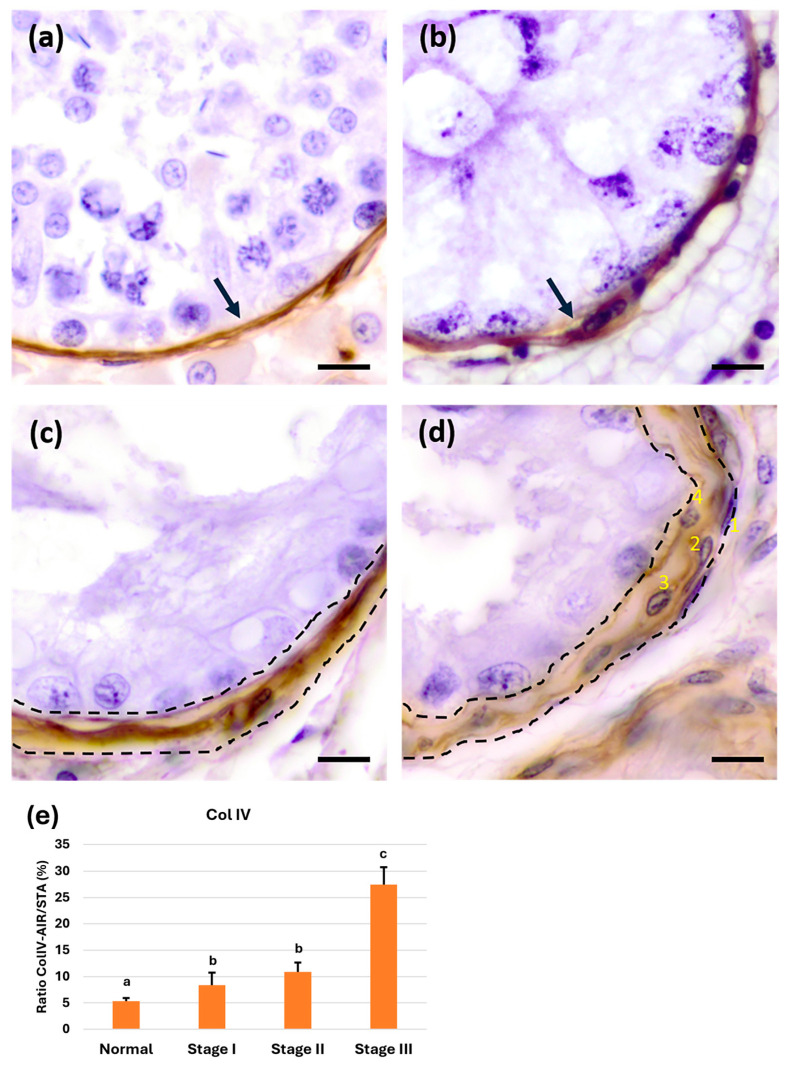
Immunohistochemical staining for collagen IV (Col IV). (**a**,**b**) Col IV immunoreactivity pattern in normal tubules (**a**) and in stage I tubular atrophy (**b**) (arrows). (**c**) In stage II, tubular wall thickening was observed, well delineated by the presence of Col IV (dotted line). (**d**) In stage III tubular atrophy, the previously described pattern of four cell layers (1 to 4) was observed, delineated by Col IV immunoreactivity in the tubular wall (dotted lines). (**e**) Semiquantitative results expressed as the Col IV-AIR/STA (%) ratio of Col IV immunoreactivity during the different stages of tubular atrophy. a, b, and c show significant differences (*p* < 0.05). Scale bar: 10 µm.

It is well established that Col IV is a main component of the BM and is not expressed by seminiferous tubule cells. From a qualitative perspective, Col IV immunoreactivity was exclusively observed in the seminiferous tubule wall across all stages of atrophy (Figure 6a–d). Notably, in stage II atrophy (Figure 6c), the PLP was strongly positive for this protein. Finally, in stage III atrophy (Figure 6d), the highest number of peritubular cell layers was observed, along with Col IV positivity.

From a semiquantitative perspective, Col IV expression in the different stages of cryptorchidism-induced testicular atrophy was quantified as the ratio between the area of Col IV immunoreactivity (Col IV-AIR) and STA. A significant increase (*p* < 0.05) in Col IV immunoreactivity was observed from the first stage of tubular atrophy (stage I), with its highest expression maintained in more advanced stages of atrophy (stage II), and significantly increasing again (*p* < 0.05) in stage III atrophy tubules (Figure 6e). These results indicate that Col IV expression increases as the cryptorchidism-induced tubular atrophy progresses, correlating with the previously observed HSP47 increase in stage II atrophy Sertoli cells and in stage III atrophy with higher expression in peritubular myoid cells.

## 4. Discussion

Immunohistochemical pattern found in healthy boar PLP is like the results found in other species with several layers of myoid cells, as is the case of human species [14]. The HSP47 protein is a chaperone directly involved in the synthesis and correct folding of collagens. It is highly expressed in cells with high collagen production, such as fibroblasts, chondroblasts, osteoblasts, and odontoblasts [37]. In the testis, its presence has been previously demonstrated in Leydig cells, myoid cells, Sertoli cells, and fibroblasts of the intertubular interstitium in rodents [34]. In this study, the presence of HSP47 in the peritubular myoid and Sertoli cells of boar testes suggests that this protein is expressed under physiological conditions in other mammals. Furthermore, in our study, HSP47 expression increased in seminiferous tubule sections that exhibited significant cellular alterations, particularly in stages II and III atrophy compared with stage I. This indicates that more severe tubular injury is likely associated with a modification of Sertoli and myoid cell phenotypes, which exhibit a fibroblastic character. A similar modification has been observed in Sertoli cells of tubule sections during spermatogenic arrest in aging hamster testes [34]. In stage II tubule sections, HSP47 expression significantly increased only in Sertoli cells and, subsequently, between stages II and III, only in myoid cells. This suggests a biphasic progression of seminiferous tubule deterioration. In the first phase, the loss of germ cells does not affect HSP47 expression in the seminiferous tubules (stage I). Subsequently, morphological changes in Sertoli cells show increased HSP47 expression, likely indicating greater Col IV synthesis (stage II). This change probably affects myoid cells, which are usually regulated by Sertoli cells, causing them to acquire a phenotype that expresses more HSP47 and is associated with the synthesis of new collagen I (stage III). Thus, tubular sclerosis and its subsequent evolution due to collagen I accumulation seem to depend on myoid cells, considering that HSP47 expression in Sertoli cells in stage III seminiferous tubules returns to control levels, indicating a possible final alteration in Sertoli cell phenotype. In summary, the results suggest that increased HSP47 expression is a good marker of collagen deposition during the tubular sclerosing process. These changes also strongly correlate with modifications in vimentin expression in Sertoli and myoid cells. Initially, vimentin expression increases in Sertoli cells and subsequently shifts to myoid cells between stages II and III, while decreasing in Sertoli cells. Changes in vimentin expression patterns in Sertoli cells have been found in various pathological situations, including cryptorchidism. Vimentin is a well-studied filament in Sertoli cells and has been implicated in the aging process, where Sertoli cells present an increased abundance of vimentin [38,39,40]. Thus, elevated vimentin levels can be a warning sign of disrupted homeostatic functions, along with increased HSP47 expression.

An immunohistochemical study showed that the vimentin-positive area in seminiferous tubule cross-sections was significantly increased in testis tissue sections of a vitamin E-deficient group compared with normal sections. The vimentin-positive Sertoli cells extended greatly from the BM, suggesting that vimentin may be a potential marker of alterations in the spermatogenic process [41]. In cryptorchidism, results are controversial. In pigs, the number of vimentin-expressing Sertoli cells does not change, and male germ cells, not Sertoli cells, are specifically damaged by heat in cryptorchid pig testes [42]. However, another study showed that vimentin in Sertoli cells of cryptorchid testes exhibits a very immature pattern at five months of age, with abnormal infranuclear vimentin extensions at eight months of age, and lower vimentin staining intensity at 18 months of age. This work concluded that cryptorchidism development can delay the peritubular myoid cell differentiation, resulting in an abnormal vimentin pattern in Sertoli cells, which appear less differentiated than in scrotal testes [43]. Other authors observed that, in the cryptorchid testis of rhesus monkeys, vimentin is localized in the perinuclear region with intense and disorganized staining, suggesting that Sertoli cells in primates can be affected by heat stress. Altered intermediate filaments could induce functional changes in Sertoli cells, contributing to germ cell apoptosis and leading to azoospermia or oligozoospermia [44]. Similar findings have been reported in rat cryptorchidism, where intense vimentin immunoreactivity surrounds Sertoli cell nuclei, along with the collapse of apical extensions. The authors proposed that Sertoli cells are affected in cryptorchidism, and altered vimentin filament distribution correlates with increased germ cell apoptosis. The vimentin filaments in Sertoli cells are important for maintaining the structural integrity of the seminiferous epithelium and preventing increased apoptosis [17]. Our results show that Sertoli cells with an altered vimentin pattern in the early stages of histological tubular alteration indicate a modification of these cells, leading to an increased fibroblastic character. Later, during the final phase, these cells lose this character. The Col IV staining pattern seems to confirm this progression of the tubular wall, as the deposition of Col IV, initially driven by HSP47 expression in Sertoli cells (stages I and II with a slight progressive increase in proteoglycan synthesis), reaches a maximum in stage III when HSP47 expression in Sertoli cells decreases. At this point, Col IV and proteoglycan production and deposition peak, and these cells appear to show less fibroblastic differentiation, as indicated by reduced vimentin positivity.

The myoid or peritubular cells of the seminiferous tubules in scrotal testes exhibit a strong affinity for vimentin and HSP47, displaying a fibroblastic character that becomes more pronounced in tubule sections with severe cryptorchid lesions. Transmission electron microscopy of boar testes has shown that peritubular cells in the innermost layer have a contractile character, while the outer cells are more fibroblastic in normal testes [19]. In cryptorchid tubules, both layers appear fibroblastic [19], which is consistent in part with our immunohistochemical findings. On the one hand, we observed that the innermost layer of myoid cells loses α-actin positivity in stage III atrophy, while the other layers exhibit a myofibroblastic nature in both normal and cryptorchidic tubules. In humans, the myoid cell phenotype in cryptorchidism is also myofibroblastic, with fibroblastic cells in the outermost layer [3]. Similar to our findings, the myofibroblast dedifferentiation is not responsible for the disturbed spermatogenesis associated with PLP alterations [3]. In our study, the presence of a new peritubular cell layer in damaged stage III tubules may be linked to the incorporation of interstitial fibroblasts into the tubular wall, where they differentiate into myofibroblasts and contribute to collagen accumulation. In summary, the tubular wall contains cells with both contractile and collagen-synthesizing characteristics. Myoid cells exhibit a mixed pattern during tubular degeneration, especially in the middle and outer zones when incorporated from the interstitium. These cells not only show fibroblastic features but also express α-actin in stage III atrophy. In contrast, the innermost layer loses contractility in the most advanced stages of atrophy, retaining only fibroblastic features. In healthy seminiferous tubules, only myofibroblast cells were present.

Collagen (I and III) deposition—evaluated using PSR staining—was greater in stage III atrophy, likely due to increased HSP47 expression in myoid cells during the final stages of tubular degeneration. These results suggest that the initial increase in PLP thickness was probably a result of collagen accumulation from the shortening and folding of the seminiferous tubules. In stages II and III, HSP47 expression leads to new collagen I and III synthesis, which accumulates in the PLP, forming a thicker lamina not associated with tubule folding.

## 5. Conclusions

In conclusion, the progression of seminiferous tubule lesions in spontaneous cryptorchidism in pigs exhibits a gradual phenotypic fibroblastic transformation in Sertoli and myoid cells. This phenomenon initially affects Sertoli cells and later myoid cells. The deposition of collagen I and III in the PLP is primarily driven by myoid cells between stage II and III atrophy, marking the onset of sclerosis and hyalinization of the seminiferous tubules. Finally, these results may be relevant in human clinical practice since the boar is widely used as animal suitable model for study of human disease [45].

## Figures and Tables

**Table 1:** Histomorphometric data of different types of seminiferous tubules in cryptorchid boar.

	Normal	Stage I	Stage II	Stage III
Tubular diameter (µm)	214.89 ± 8.89 ^a^	111.29 ± 1.38 ^b^	148.80 ± 3.69 ^c^	77.03 ± 2.55 ^d^
Lumen diameter (µm)	85.44 ± 9.15 ^a^	30.43 ± 6.69 ^b^	107.73 ± 5.26 ^a^	16.21 ± 1.69 ^b^
Epithelial height (µm)	64.72 ± 3.13 ^a^	51.90 ± 0.99 ^a^	20.52 ± 2.06 ^b^	23.83 ± 1.91 ^b^

a, b, c, and d represent significant differences between stages when *p* value < 0.05.

## Data Availability

All data provided have been extracted from the various articles and abstracts consulted.
